# The first comprehensive stroke unit was established in Iran

**Published:** 2013

**Authors:** Masoud Mehrpour

**Affiliations:** Assistant Professor, Department of Neurology AND Stroke Center, Firoozgar General Hospital, Iran University of Medical Sciences, Tehran, Iran

The burden of stroke is unarguably a national issue and should be addressed by the government. Stroke Units are wards dedicated to the care of acute stroke patients and stroke patients who need rehabilitation by a multidisciplinary team of health professionals, who are trained specifically in the care of stroke patients. There is evidence that organized multidisciplinary care is more effective than care in a general medical ward.

Despite successes in inventing effective new therapies, significant obstacles remain in providing them to the patients. What Iran needs to optimize stroke care is to acknowledge the problem and flaws in the existing system, vision, initiation, and guidance. In many instances, these obstacles may be related to a fragmentation of coordination of important stakeholders of stroke care. Auspiciously, since 2 years ago, we have established the first primary stroke unit in Iran. During this time, we have treated more than 40 acute stroke patients with intravenous rtPA. In the tradition of providing superior medical care, the neurology department of Firoozgar Academic Hospital, affiliated to Tehran University of Medical Sciences (TUMS), has inaugurated a new comprehensive stroke unit, the first of its kind in the region. This unite is dedicated to providing around-the-clock assessment, diagnosis, and definitive treatment for those who suffer from all types of strokes. Firoozgar Hospital is located near Valiasr Square in the center of Tehran.

The main goals of this Intensive Stroke Unit are to improve the stroke treatment standards in Iran and the region, and to be the leader in stroke therapy.

Firoozgar Comprehensive Stroke Center is comprised of specialized physicians and nurses, advanced imaging modalities, up-to-date medical treatment (including thrombolysis; intravenous or intra-arterial clot dissolving agents, thrombectomy, angioplasty), and post-stroke rehabilitation services.

**Figure 1:**
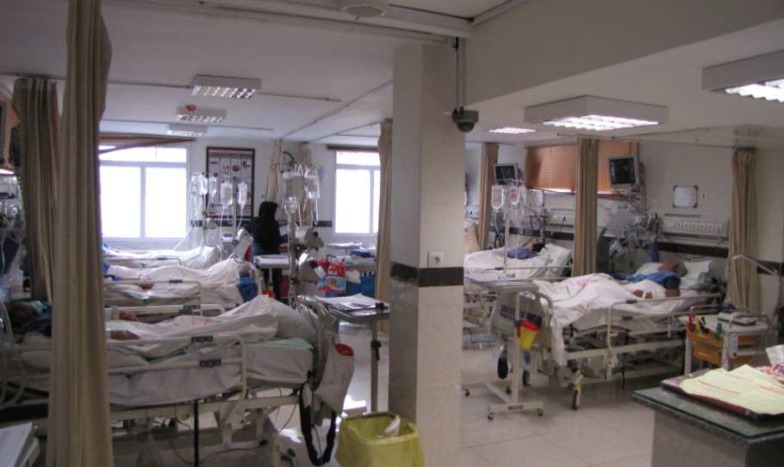
Firoozgar stroke unit with 8 beds and facilities needed for caring for patients suffering from acute stroke

**Figure 2:**
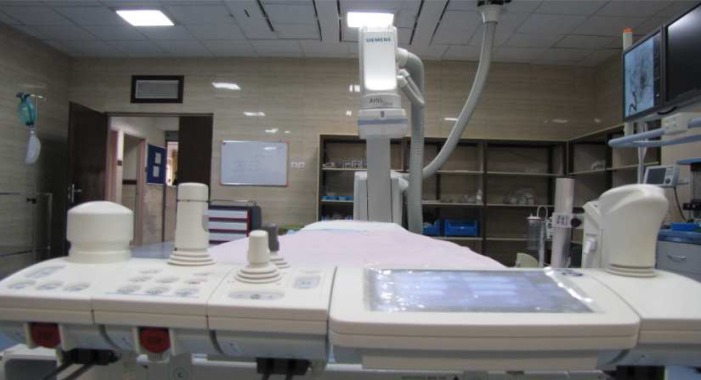
The Angiography Unit is ready around the clock for performing neurointervention, including intra-arterial thrombolysis, embolectomy, and angioplasty

In our stroke center, we have 8 beds with all the facilities needed for acute stroke, and 22 beds in the post-stroke ward (Figure 1). The Angiography Unit is ready around the clock for performing neurointervention, including intra-arterial thrombolysis, embolectomy, and angioplasty (Figure 2).

In the near future, we hope to organize a stroke unit network in different districts of Tehran to provide a valuable facility for stroke patients.

